# Are Antioxidants Useful in Preventing the Progression of Chronic Kidney Disease?

**DOI:** 10.3390/antiox10111669

**Published:** 2021-10-23

**Authors:** Alfredo G. Casanova, Francisco J. López-Hernández, Laura Vicente-Vicente, Ana I. Morales

**Affiliations:** 1 Institute of Biomedical Research of Salamanca (IBSAL), 37007 Salamanca, Spain; alfredogcp@usal.es (A.G.C.); flopezher@usal.es (F.J.L.-H.); amorales@usal.es (A.I.M.); 2 Toxicology Unit, University of Salamanca (USAL), 37007 Salamanca, Spain; 3 Department of Physiology and Pharmacology, University of Salamanca (USAL), 37007 Salamanca, Spain; 4 Group of Translational Research on Renal and Cardiovascular Diseases (TRECARD), 37007 Salamanca, Spain; 5 National Network for Kidney Research REDINREN, Instituto de Salud Carlos III, 28029 Madrid, Spain; 6 Group of Biomedical Research on Critical Care (BioCritic), 47005 Valladolid, Spain

**Keywords:** chronic kidney disease, antioxidants, meta-analysis, bardoxolone methyl, pentoxifylline, nephroprotection

## Abstract

Chronic kidney disease (CKD) is a progressive impairment of renal function for more than three months that affects 15% of the adult population. Because oxidative stress is involved in its pathogenesis, antioxidants are under study for the prophylaxis of CKD progression. The objective of this work was to meta-analyze the efficacy of antioxidant therapy in CKD patients and to identify the most effective candidate antioxidants. Our meta-analysis showed that, despite being quite heterogeneous, overall antioxidant therapy apparently reduced CKD progression. Pentoxifylline and bardoxolone methyl demonstrated a robust and statistically significant protection, while other products showed a favorable but non-significant tendency, due to a high interindividual variability. Off-target (i.e., antioxidant-independent) effects, such as body weight reduction and heart failure-associated blood dilution, might totally or partially explain the protection provided by effective antioxidants. This potential pleiotropy introduces uncertainty on the role of oxidative stress in CKD progression and on antioxidant therapy in its prevention, which needs to be further investigated. Independently, identification of factors determining the nephroprotective effect of each candidate on each patient is thus necessary for a prospectively personalized antioxidant therapy. Finally, pentoxifylline should be further explored for the prophylaxis of CKD progression.

## 1. Introduction

Chronic kidney disease (CKD) is a functional or structural deterioration of the kidneys lasting at least three months. The detection and progression of CKD is generally determined through the glomerular filtration rate (GFR), but also through plasma creatinine (Cr_pl_) and albuminuria [1]. According to the Kidney Disease Outcomes Quality Initiative, CKD progression is stratified into five stages (G1–G5) based on the estimated GFR (eGFR, mL/min/1.73 m^2^). Stage 1 represents early stages of chronic kidney damage (eGFR ≥ 90); stage 2, mild decrease (eGFR 60–89); stage 3, moderate to severe decrease (eGFR 30–59); stage 4 or pre-end-stage renal disease, severe decrease (eGFR 15–29); and stage 5, kidney failure (eGFR < 15) [2]. Progression beyond stages 4–5 leads to end-stage renal disease, a condition incompatible with life, in which dialysis or renal transplantation is necessary [3].

The main risk factors for CKD are hypertension, diabetes, dyslipidemia, advanced age and, to a lesser degree obesity or exposure to toxins, drugs, or heavy metals [1,4]. The aging of the population and the high prevalence of risk factors have contributed to increasing the number of people affected by CKD. This makes it a pathology with a high incidence (15% of Americans adults) [5]. It has been estimated that CKD will be the fifth leading cause of death by 2040 [1].

CKD carries a high socioeconomic burden, representing 2–3% of annual health expenditure in high-income countries [6]. In 2018, in the United States, CKD costs USD 84 billion, a budget that increases by USD 36.6 billion more when patients with end-stage renal disease (dialysis or kidney transplant) are included [5].

Renal function regulation is highly influenced by oxygen free radicals, which make the kidney very susceptible to redox imbalances and oxidative stress. It has been postulated that during the development of CKD, kidney cells are unable to correctly handle the excess in oxidative substances and undergo apoptosis and senescence [7]. The continuity of this effect over time leads to a decrease in the capacity for cell regeneration by the kidney and ultimately leads to kidney fibrosis [8]. This CKD–oxidative stress association has been shown in all CKD stages after observing (i) increases in oxidative stress biomarkers such as oxidized low-density lipoprotein, oxidized thiol compounds, and malondialdehyde; (ii) increase in biomarkers related to DNA damage by ROS such as 8-hydroxydeoxyguanosine and 8-oxodeoxyguanosine; and (iii) deterioration in antioxidant defense due to the inability to eliminate ROS, through alteration in the activity of the enzymes superoxide dismutase, myeloperoxidase, xanthine oxidase, heme-oxidase, glutathione peroxidase, or catalase [9,10]. The appearance of high amounts of uremic toxins in CKD patients could be added as another source of oxidative stress. The synthesis of uric acid promotes the activity of the oxidizing enzyme xanthine oxidase [11]. In animal models, an implication of the renin angiotensin system in oxidative stress has also been proposed. After activating the AT1 receptors in rats with CKD, higher levels of superoxide were observed compared to the control group [3].

The sum of the health and socioeconomic aspects confirms the need to find strategies that slow the progression of CKD. Given the involvement of oxidative stress in the progression of this pathology, the use of antioxidants has been extensively evaluated as a preventive strategy [7,9]. The results obtained in this field have been very diverse, and not all therapies have been as effective as prophylactic measures. Many of the antioxidants evaluated, in turn, have therapeutic properties against comorbidities (antihypertensive, antigout). This scenario could lead to a misinterpretation of the results since prevention could be more related to the improvement of the underlying pathology rather than to the antioxidant effect. To avoid this, an antioxidant–renoprotection association in CKD patients should be defined taking into account only those products that are administered as protectors without any other pharmacological objective.

Thus, this work aimed to evaluate whether the administration of antioxidants (without any other pharmacological function) is an effective strategy to slow CKD progression, and to identify those that have shown the best results in clinical studies. In this work, the term “antioxidant” refers to a product of any kind, whether natural or synthetic, that reduces oxidative stress by means of direct or indirect mechanism.

## 2. Materials and Methods

### 2.1. Retrieval of Published Studies

A bibliographic search of clinical studies published in the databases MEDLINE and ScienceDirect up to February 2021 was carried out by entering the following keyword combinations: “(Antioxidant OR Antioxidants) AND (Chronic kidney disease OR CKD)” (filters: “Clinical Trial” and “Randomized Controlled Trial”). After identifying the compounds most used in these studies, an additional search was carried out in order to find some articles not detected in the initial search, using the following combination of keywords: “(Chronic kidney disease OR CKD) AND (Allopurinol OR Amlodipin OR Arginine OR Bardoxolone OR Candesartan OR Ezetimibe OR Pravastatin OR Rosuvastatin OR Selenium OR Simvastatin OR Sodium bicarbonate OR Valsartan OR Vitamin C OR Vitamin D OR Vitamin E).

### 2.2. Exclusion and Inclusion Criteria

Two researchers (A.G.C. and L.V.-V.) independently removed papers that met any of the following exclusion criteria: (1) reviews and protocols, (2) only abstract available, (3) unrelated content, and (4) language other than English. After that, they selected those studies that met the following inclusion criteria: (1) randomized studies where the nephroprotective efficacy of a compound is evaluated in patients with CKD; (2) studies in which the compound is administered as a supplement, i.e., not to treat any of the pathologies that the patients already presented; (3) studies that evaluate renal function of patients in terms of eGFR, Cr_pl_, or albuminuria (reporting the mean and a measure of dispersion that allows calculating the standard deviation); and (4) studies that include a control or placebo group. The articles selected by both researchers were shared, and if there was any difference in the selection of articles, this was resolved by a third researcher (A.I.M.).

### 2.3. Data Extraction

The following data were extracted from each included work: name of the first author and year of publication, study design, location, total duration of the study, characteristics of the patients included, nephroprotector (s) administered, daily dose, route of administration, and duration of treatment. Clinical study design quality was calculated according to the Jadad scale [12]. Studies with a score of 5 were considered rigorous, scores between 3 and 5 were considered good quality, and scores below 3 were considered poor quality. Additionally, the mean and standard deviation (SD) values of the parameters eGFR, Cr_pl_, and/or albuminuria were registered (or calculated from the standard error of the mean or the confidence interval). From these numerical data, the mean increase in each biomarker (*BM*_Δ_) of renal function was calculated in the treated groups and in the control/placebo groups with the formula: *BM*_Δ_ = *BM*_F_ − *BM*_B_, where *BM*_F_ is the mean value of the biomarker at the end of the nephroprotective treatment, and *BM*_B_ is the mean baseline value of the biomarker. The standard deviation resulting from this difference, *s*_Δ_, was also calculated as the accumulation of errors: $s_{\Delta }=\sqrt{s_{F}{}^{2}+s_{B}{}^{2}}$, where *s_F_* is the SD value of the biomarker at the end of the nephroprotective treatment, and *s_B_* is the SD value of the biomarker at baseline.

### 2.4. Meta-Analysis

Heterogeneity between studies was assessed by applying the chi-square Q-test under the null hypothesis of homogeneity (*p* < 0.05 indicated heterogeneity) and calculating the I^2^ parameter (I^2^ > 50% indicated high heterogeneity). After this, the fixed-effects model (for homogeneous studies) or the random-effects model (for heterogeneous studies) was selected to meta-analyze the data. The Hedges’ g value and its 95% confidence interval were calculated for each study and each renal function biomarker with the following formula:

$$g=\frac{BM_{\Delta T}-BM_{\Delta C/P}}{sp};$$

where:

$$sp=\sqrt{\frac{\left(n_{T}-1\right)s_{\Delta T}^{2}+\left(n_{C/P}-1\right)s_{\Delta C/P}^{2}}{\left(n_{T}-1\right)+\left(n_{C/P}-1\right)}};$$

where *BM*_Δ*T*_ and *BM*_Δ*C/P*_ are the biomarker increases in the treatment and in the control/placebo groups, respectively; $s_{\Delta T}^{2}\;$and $s_{\Delta C/P}^{2}$ are the standard deviations of the treatment and the control/placebo groups, respectively; and $n_{T}$ and $n_{C/P}$ correspond to the sizes of the treatment and control/placebo groups, respectively. Forest plots were constructed in which the g parameters of the different included studies were compared.

Finally, funnel plots in which the Hedges’ g of each study was represented versus its standard error were constructed to evaluate potential publication bias. In addition, the asymmetry tests of Begg and Mazumdar [13] and Egger et al. [14] were applied. All the analyses described in this section were carried out with the *Meta-Essentials* set of workbooks [15].

## 3. Results

### 3.1. Data Mining

**Figure 1:**
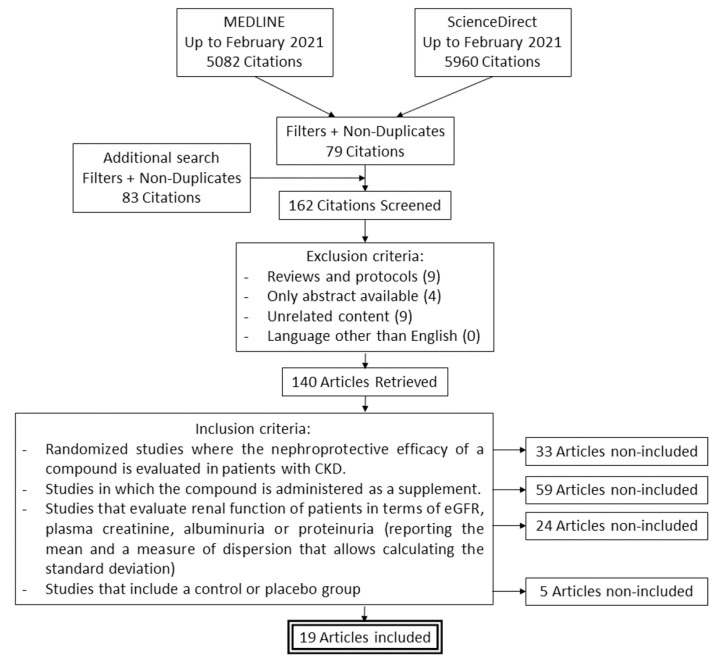
Study search and selection flow diagram made in accordance with the PRISMA guidelines.

The flow chart describing the study search process and definitive inclusion of cited references is presented in Figure 1. After the initial and additional search, 162 potential articles were identified which, after applying the exclusion and inclusion criteria, were reduced to 19. Most of the studies could not be included because they did not evaluate the improvement in renal function of the patients, administered the nephroprotective compound as part of the therapy against other secondary pathologies, or did not provide the necessary numerical data to perform the meta-analysis. The descriptive data extracted from the 19 clinical studies definitively included are shown in Table 1.

All the included studies were prospective and randomized, and most included a double-blind design. All included studies reached a Jadad score between 3 and 5 (good quality design), so absence of bias in study methodology was assumed. Seven of the studies included patients with CKD and type 2 diabetes mellitus as a specific study population. It should also be noted that almost all the studies indicated that the antioxidant treatment was administered orally throughout the study. The duration of these treatments ranged from 8 weeks to 5 years. The most widely quantified parameter to evaluate the renal function of the patients was eGFR.

### 3.2. Evaluation of the Nephroprotective Effect of the Antioxidant Compounds Tested

**Figure 2:**
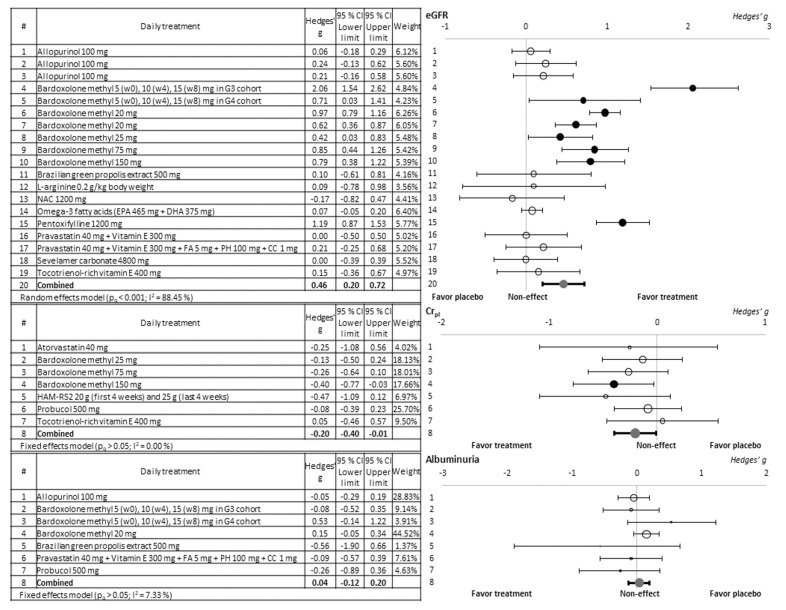
Forest plots in which the nephroprotection of the different treatments tested against CKD is compared to the placebo/control groups evaluated by means of different biomarkers of renal function. The effect size is measured as Hedges’ g ± 95% CI. CC: cyanocobalamin; CI: confidence interval; Cr_pl_: plasma creatinine; DHA: docosahexaenoic acid; eGFR: estimated glomerular filtration rate; EPA: eicosapentaenoic acid; FA: folic acid; NAC: N-acetylcysteine; PH: pyridoxine hydrochloride; w: week.

The meta-analytical evaluation of the different antioxidant supplements tested against CKD in terms of improvement of eGFR, Cr_pl_, and albuminuria is presented in Figure 2.

As observed in the forest plot, only two of the antioxidant compounds evaluated, bardoxolone methyl (in all the doses tested) and pentoxifylline, managed to cause a significant improvement in the parameter eGFR of the treated patients. Out of the articles including methyl bardoxolone, one [25] showed a substantially higher Hedges’ g when bardoxolone was specifically administered to patients with stage 3 CKD. However, when bardoxolone was administered to stage 4 patients in the same study, the Hedge’s g remained lower, i.e., at the level shown by the other studies of bardoxolone. Regarding the rest of the products, the high variability of the effect observed in them does not allow significant results to be obtained. On the other hand, a beneficial effect was only detected from the highest tested dose of bardoxolone methyl in terms of the Cr_pl_ biomarker, and no significant protective effect of the albuminuria parameter was observed.

### 3.3. Assessment of Publication Bias

**Figure 3:**
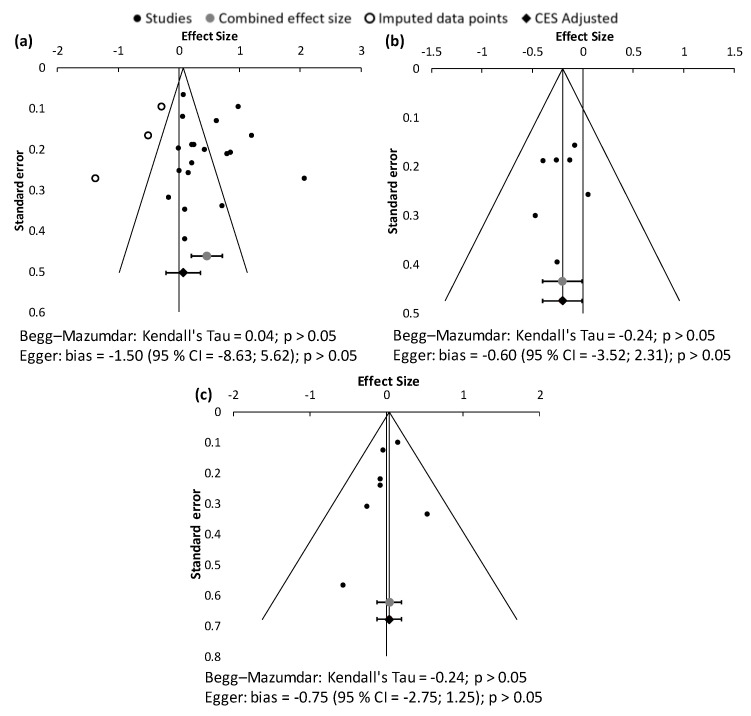
Funnel plots and asymmetry tests corresponding to the meta-analysis of the parameters eGFR (**a**), Cr_pl_ (**b**), and albuminuria (**c**). The effect size is measured as Hedges’ g ± 95% CI. CES: combined effect size; CI: confidence interval.

As described in Section 2.4, the existence of publication bias was evaluated graphically with the elaboration of funnel plots and numerically with the application of asymmetry tests. The results of this analysis are shown in Figure 3. Despite observing a slight asymmetric trend in the funnel plot corresponding to the eGFR parameter, none of the statistical tests applied detected a significant degree of asymmetry. Therefore, it can be assumed that in this meta-analysis work there is no high publication bias.

## 4. Discussion

The potential utility of antioxidants for slowing CKD progression has been proposed independently by different studies reporting the beneficial effects of candidate products. In this article, we conducted a meta-analysis to obtain a pooled, integrated, and statistically driven view. As a novelty in this work, studies reporting antioxidants administered specifically to alleviate CKD-inducing comorbidities (e.g., antidiabetics, antihypertensives) were excluded as their protective effect could be falsely attributed to their antioxidant properties. At first glance, our results indicate that antioxidants significantly reduce the decline in eGFR while having no effect on Cr_pl_ or albuminuria. During CKD, albuminuria is reflective of deterioration of the glomerular filtration barrier or of defective tubular reabsorption. It would thus be possible that antioxidants exerted a rather differentiated effect on specific renal processes. By contrast, as a surrogate of GFR, Cr_pl_ would be expected to show a similar profile.

However, a deeper insight suggests that the overall effect on eGFR is mostly due to the influence of two products (i.e., pentoxifylline and bardoxolone methyl), with the other antioxidants showing no individual or composite benefit. Because pentoxifylline and bardoxolone behave dissimilarly to the other candidates, their protective effects could be unrelated to their antioxidant properties. If they were excluded from the meta-analysis, the overall effect on eGFR would be more similar to the results for Cr_pl_ This analysis questions the efficacy of antioxidants at ameliorating glomerular filtration decline and GFR progression. Pentoxifylline and methylbardoxolone are not intrinsically antioxidant chemicals. Their antioxidant effect results from the inhibition of signaling pathways leading to oxidative stress. In fact, pentoxifylline and bardoxolone hoard additional properties that might explain their differential efficacy.

Pentoxifylline is a phosphodiesterase inhibitor with antioxidant, antifibrogenic, anti-inflammatory, and immunomodulatory properties [35], which are especially relevant as renal interstitial fibrosis and inflammation are main progression factors of CKD [36,37,38]. Of note, pentoxifylline reduces proteinuria and albuminuria in diabetic kidney disease more effectively in advanced stages of CKD [39,40]. This benefit has been associated with milder inflammation and lower levels of pro-inflammatory cytokines, both in patients [41] and in an animal model of diabetic nephropathy [42,43,44]. Interestingly, pentoxifylline has not shown significant adverse effects [44]. However, the role of this antioxidant activity as a protective mechanism has been questioned, as Zhang et al. (2016) reported renal improvement without changes in oxidative stress [35].

Bardoxolone methyl, an orally bioavailable semisynthetic triterpenoid, not only prevents renal deterioration but even improves eGFR [45], probably due to its ability to maintain redox balance and to its cytoprotective effect [46,47]. Its clinical utility remains in doubt, with it being associated with an increase in albuminuria (a known predictor of kidney disease progression) [48], which leads to the debate as to whether bardoxolone methyl really protects against CKD or if it only exerts a delusive effect by directly increasing GFR [45,49]. Other effects reported with this drug have been body weight reduction [25], and increased heart failure, hospitalizations, and mortality [48]. These effects were detected in a phase 3 clinical trial evaluating this product for the treatment of chronic kidney disease (CKD) [34]. Higher mortality, glomerulosclerosis, and tubular alterations have also been observed in diabetic Zucker rats [50].

Conclusions are also limited by the absence of oxidative stress markers in most studies, which makes it difficult to associate renal protection with oxidative stress. Of the 18 articles included, only one evaluated plasma malondialdehyde [33] and two others also measured plasma-oxidized low-density lipoprotein [16,24]. Their results are heterogeneous: (i) slight renal protection associated with a decrease in oxidative stress [33], (ii) no renal protection along with no modification of oxidative stress markers [24], and (iii) no improvement in kidney function despite a net reduction in oxidative stress [16]. In addition, the sample size was insufficient to reach robust conclusions in all cases. Future studies would benefit from including oxidative stress markers along with renal function evaluation.

**Figure 4:**
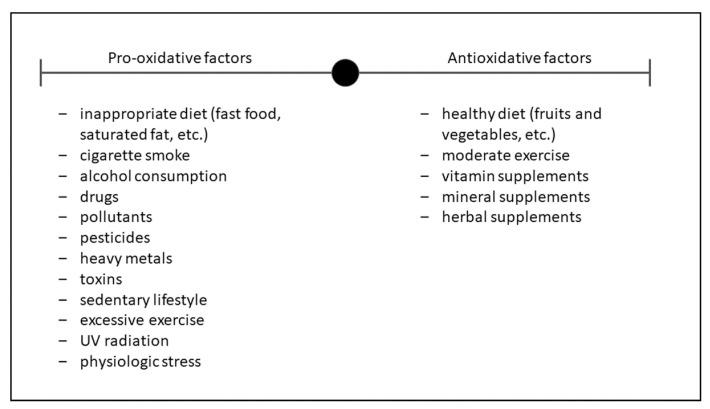
Influence of lifestyle on oxidative stress. On the left side, the factors that favor the oxidative state are listed, while on the right side are those that favor antioxidant activity.

Another finding worth mentioning is the high internal variability observed in many studies, which is inferred from their large error bars and implies a very heterogeneous response from patient to patient. The location of the bars in the plot significantly crossing the no-effect line reveals that this heterogeneity ranges from patients who respond to specific antioxidants to those who do not, and even others showing impairment in renal function. The potential causes of variability are varied, including diverse adherence to the antioxidant therapy. Renal function prior to treatment inception may impact outcome, as antioxidants have been shown to be more effective in advanced stages of CKD [51]. Pro-oxidant and antioxidant lifestyles (Figure 4), comorbidities, and patient characteristics would pose additional sources of variability [52,53]. Should this be so, certain individuals could benefit from specific antioxidants and a personalized antioxidant therapy might thus be envisaged.

This concept has been recently proposed in the field of oxidative stress associated with exercise and insulin resistance. A “redox screening” based on clinical evidence has been delineated, which integrates information on the individual and candidate antioxidants to characterize different profiles based on the specific deficiency in the physiological redox regulation and thus apply the most effective antioxidant treatment [54]. In fact, it was previously shown that, e.g., N-acetylcysteine (NAC) supplementation was only effective in improving physical performance in individuals with low glutathion levels; whereas vitamin C supplementation was only effective in vitamin C-deficient individuals [55]. A similar strategy may be generated with a CKD scope. For this purpose, new clinical trials with specific designs are necessary. Besides a coherent sample size, these new trials should test different doses of the antioxidant candidate and, most importantly, collect matched information on renal function and redox status, lifestyle, and comorbidities. Artificial-intelligence-driven algorithms may be built to aid in the selection of the most appropriate antioxidant therapy for each patient, based on the integration of individual clinical data.

Prospectively, in alignment with the emerging concept of precision medicine, the individual level, type, and source of oxidative stress may be used first to further study the relation of oxidative stress and CKD progression, and then to guide decision making in relation to the prevention or treatment of this disease with a personalized antioxidant therapy.

## 5. Conclusions

The main conclusion of this study is that an absence of robust data to either support or dismiss the hypothesis that inhibition of oxidative stress slows or ameliorates CKD progression has been determined. As a corollary, the role of oxidative stress in CKD progression remains uncertain, despite several authors having suggested its participation in CKD pathophysiology [9,54,56]. Future studies need to be optimized with matched data from renal function and oxidative stress. An identification of the factors determining the nephroprotective efficacy of each antioxidant candidate on each patient is also necessary for a prospectively personalized therapy. Regardless of the mechanism involved, this meta-analysis identified pentoxifylline as a potentially interesting candidate for the prophylaxis of CKD progression, which merits further development.

## Figures and Tables

**Table 1:** Descriptive characteristics of the studies included in the meta-analysis. AU: albuminuria; Cr_pl_: plasma creatinine; eGFR: estimated glomerular filtration rate; p.o.: per os (oral administration).

Study Identification	Design	Location	Duration of Recruitment	Population	Number of Patients Initially Included (Treatment/Control or Placebo Group)	Nephroprotective Treatment	Jadad Score	Renal Function Biomarkers Available		
								eGFR	Cr_pl_	AU
Adema et al., 2016 [16]	Prospective, randomized, double-blind trial	The Netherlands	May 2001–December 2002	Non-diabetic patients with mild–moderate chronic renal failure who had no manifest arterial occlusive disease	34/28	Pravastatin 40 mg/day (from baseline) + Vitamin E 300 mg/day (from month 6) p.o. for 12 months	5	Yes	No	No
Badve et al., 2020 [17]	Prospective, randomized, double-blind trial	Australia and New Zealand	March 2014–December 2016	Adults with stage 3 or 4 chronic kidney disease and no history of gout	182/181	Allopurinol 100 mg/day (the first 12 weeks) and up to 300 mg/day (until the end of the study) p.o. for 2 years	4	Yes	No	Yes
de Boer et al., 2019 [18]	Prospective, randomized, double-blind trial	USA	November 2011–March 2014	Patients with type 2 diabetes at baseline to ascertain CKD outcomes	289/320	Omega-3 fatty acids (Eicosapentaenoic acid 465 mg/day + Docosahexaenoic acid 375 mg/day) p.o. for 5 years	5	Yes	No	No
Chin et al., 2018 [19]	Prospective, randomized, double-blind trial	USA, European Union, Australia, Canada, Israel, and Mexico	June 2011–September 2012	Patients with type 2 diabetes mellitus and stage 4 chronic kidney disease	1097/1088	Bardoxolone methyl 20 mg/day p.o. for 48 weeks	4	Yes	No	No
Endo et al., 2013 [20]	Prospective, randomized, open-label trial	Japan	October 2001–September 2004	Patients with type 2 diabetes mellitus and albuminuria	80/82	Probucol 500 mg/day for 5 years	3	No	Yes	Yes
Goicoechea et al., 2010 [21]	Prospective, randomized, open-label trial	Spain	January 2007–May 2007	Patients with chronic kidney disease	57/56	Allopurinol 100 mg/day for 24 months	5	Yes	No	No
Goicoechea et al., 2015 [22]	Prospective, randomized, open-label trial	Spain	May 2007–May 2012	Patients with chronic kidney disease	57/56	Allopurinol 100 mg/day for 5 years	5	Yes	No	No
Koay et al., 2021 [23]	Prospective, randomized, double-blind trial	Malaysia	March 2019–September 2020	Patients with diabetic kidney disease	31/30	Tocotrienol-rich vitamin E 400 mg/day p.o. for 12 months	4	Yes	Yes	No
Nanayakkara et al., 2007 [24]	Prospective, randomized, double-blind trial	The Netherlands	May 2001–December 2002	Non-diabetic patients with chronic renal failure who had no manifest arterial occlusive disease	47/46	Pravastatin 40 mg/day (from baseline) + Vitamin E 300 mg/day (from month 6) + Folic acid 5 mg/day, pyridoxine hydrochloride 100 mg/day and cyanocobalamin 1 mg/day (from month 12) p.o. for 24 months	5	Yes	No	Yes
Nangaku et al., 2020 [25]	Prospective, randomized, double-blind trial	Japan	December 2014–September 2017	Patients with with type 2 diabetes and stage 3 (cohort G3) and stage 4 CKD (cohort G4)	41/41 (G3) 24/14 (G4)	Bardoxolone methyl mg/day, followed by dose escalation, as tolerated, to 10 mg/day at week 4 and 15 mg/day at week 8 p.o. for 8 weeks	4	Yes	No	Yes
Navarro-González et al., 2015 [26]	Prospective, randomized, open-label trial	Spain	January 2008–December 2008	Patients with type 2 diabetes mellitus and diabe tic nephropathy	82/87	Pentoxifylline 1200 mg/day p.o. for 2 years	4	Yes	No	No
de Nicola et al., 1999 [27]	Prospective, randomized, double-blind trial	Italy	Not specified	Patients with proteinuria aged 18 to 60 years with a moderate to medium degree of chronic renal failure	11/10	L-arginine 0.2 g/kg body weight/day p.o. for 6 months	3	Yes	No	No
Pergola et al., 2011 [28]	Prospective, randomized, double-blind trial	USA	Not specified	Adults with moderate to severe CKD and type 2 diabetes	57/57/56/57	Bardoxolone methyl 25 mg/day or Bardoxolone methyl 75 mg/day or Bardoxolone methyl 150 mg/day p.o. for 52 weeks	4	Yes	Yes	No
Renke et al., 2008 [29]	Prospective, randomized, open-label, cross-over trial	Poland	Not specified	Non-diabetic patients aged 18 to 65 with chronic kidney disease	20/20	N-acetylcysteine 1200 mg/day p.o. for 8 weeks	3	Yes	No	No
Renke et al., 2010 [30]	Prospective, randomized, cross-over trial	Poland	Not specified	Non-diabetic patients aged 18 to 65 with chronic kidney disease	14/14	Atorvastatin 40 mg/day p.o. for 12 weeks	3	No	Yes	No
Ruggiero et al., 2019 [31]	Prospective, randomized, open-label, cross-over trial	Italy	November 2013–December 2014	Adults with CKD and proteinuria	53/53	Sevelamer carbonate 4800 mg/day p.o. for 3 months	3	Yes	No	No
Silveira et al., 2019 [32]	Prospective, randomized, double-blind trial	Brazil	Not specified	Patients aged 18 to 90 with chronic kidney disease and proteinuria	18/16	Brazilian green propolis extract 500 mg/day (35.5 mg total flavonoids + 77.96 mg total phenolic compounds) p.o. for 12 months	5	Yes	No	Yes
Tayebi-Khosroshahi et al., 2018 [33]	Prospective, randomized, double-blind, parallel trial	Iran	November 2016–February 2017	End-stage renal disease patients maintained on chronic hemodialysis	22/22	HAM-RS2 20 g/day (the first 4 weeks) and 25 g/day (until the end of the study) p.o. for 8 weeks	4	No	Yes	No
Zeeuw et al., 2013 [34]	Prospective, randomized, double-blind, parallel trial	USA, European Union, Australia, Canada, Israel, and Mexico	June 2011–September 2012	Patients with type 2 diabetes mellitus and stage 4 chronic kidney disease	1088/1097	Bardoxolone methyl 20 mg/day p.o. for 56 weeks	4	Yes	No	Yes
